# Specialist clinical pathways in audiology services for adults living with coexisting hearing loss and dementia: a scoping review protocol

**DOI:** 10.1136/bmjopen-2024-087418

**Published:** 2024-12-26

**Authors:** Sophie Mai Wenje, Sian Calvert, Helen Henshaw, Ruth V Spriggs, Tom Dening, Elizabeth Hendron, Eithne Heffernan

**Affiliations:** 1 Hearing Sciences, Mental Health and Clinical Neurosciences, School of Medicine, University of Nottingham, Nottingham, UK; 2 National Institute for Health and Care Research (NIHR) Nottingham Biomedical Research Centre, Nottingham, UK; 3 Mental Health and Clinical Neurosciences, School of Medicine, University of Nottingham, Nottingham, UK; 4 Library, Nottingham University Hospitals NHS Trust, Nottingham, UK

**Keywords:** Dementia, Review, Audiology, Cognition

## Abstract

**ABSTRACT:**

**Introduction:**

Both hearing loss and dementia are associated with ageing, and it is thought that many individuals living with dementia also live with hearing loss. Despite the large comorbidity between these two disorders, there remains a clear lack of established guidelines in audiological services for assessing and managing patients living with dementia. This scoping review aims to examine whether specialist clinical pathways exist in audiology services for people living with coexisting hearing loss and dementia and to describe the specific components and features of these pathways. This review will provide up-to-date information on clinical practice, identifying any gaps in care and in the literature to inform future research hypotheses and best practice guidelines.

**Methods and analysis:**

The methods are reported according to the Preferred Reporting Items for Systematic Reviews and Meta-Analyses extension for Scoping Reviews. The following electronic databases will be searched: CINAHL, EMBASE, MEDLINE, PsycINFO, PubMed, Scopus and Web of Science. The eligibility criteria are defined according to the domains of the SPIDER (Sample, Phenomenon of Interest, Design, Evaluation and Research type) search strategy tool. Primary research studies and select grey literature sources (eg, practice guidelines) will be eligible if published within the last 15 years. Studies eligible for inclusion must contain adults living with suspected or confirmed dementia, their carers, or clinicians within audiology services. Initial searches were performed on 31 January 2024 and will be updated before completion and submission of the review. Article quality will be appraised using an established tool: the Mixed Methods Appraisal Tool. The results will be synthesised and reported in line with reflexive thematic analysis guidelines.

**Ethics and dissemination:**

No ethical issues are foreseen as the review will collect secondary data only. Findings will be reported by peer-reviewed publication and by national and international academic conferences.

**STRENGTHS AND LIMITATIONS OF THIS STUDY**

- A strength of this research is that patient and public involvement contributors will contribute to each key stage, including data interpretation, to ensure the review is inclusive of their perspectives and priorities.
- Selected grey literature (including national or international practice guidelines) will be included so that a wide range of important sources of information about current practice will be reviewed.
- This research will be reported in accordance with the Preferred Reporting Items for Systematic Reviews and Meta-Analyses extension for Scoping Reviews checklist to ensure its transparency and completeness.
- A limitation of this study is that only literature available in English is eligible for inclusion, which may exclude some international current practice from being reviewed.

## Introduction

 Hearing loss is a major healthcare burden, which increases in prevalence and severity with age.^1^ Globally, over 5% of the population have disabling hearing loss, and by 2050, this figure is estimated to rise to 10%.^2^ In the United Kingdom (UK), as many as one in three adults are estimated to experience hearing loss.^3^ This condition can have a substantial impact on psychological, social and occupational well-being.^4^ Common experiences include difficulty maintaining concentration and participation in conversation, diminished social relationships and stigma.^5^ Hearing loss can also cause ‘third-party disability’, which has been defined as the referred disability of family members due to the health condition of their significant other.^4 6^ Furthermore, hearing loss is a potential risk factor for the development of dementia.^7^

Globally, more than 55 million people live with dementia, including over 900 000 people within the UK.^8 9^ Dementia is a clinical syndrome that can be caused by several progressive diseases that damage the brain and destroy nerve cells, which leads to a deterioration in cognitive function and changes in mood and behaviour.^8 10 11^ The most common form of dementia is Alzheimer’s disease (AD), which accounts for 60–80% of cases and is associated with amyloid and tau pathology within the brain.^12^ Other forms of dementia include vascular dementia, dementia with Lewy bodies and frontotemporal dementia. It can also be associated with certain neurological disorders (eg, Parkinson’s disease) and infections (eg, HIV), harmful alcohol use and repetitive physical injuries to the brain.^8 13 14^ Dementia is characterised by severe cognitive impairments, particularly memory problems, and functional impairments or difficulty with activities of daily living. It can cause problems with planning, decision making, communication, hallucinations, apathy, depression and social withdrawal.^8 10 11^ Mild cognitive impairment (MCI) is a heterogeneous clinical syndrome in which people experience a degree of cognitive decline that is not normal for their age group but without the substantial impairment in daily functioning that is required for the diagnosis of dementia.^15^,^17^ Amnestic MCI primarily impacts memory, while non-amnestic MCI affects other aspects of cognition (eg, decision making, visual perception).^18^ People living with MCI have a higher risk of developing dementia than age-matched peers. Studies suggest that approximately 15% develop dementia after 2 years and about one-third develop dementia due to Alzheimer’s disease within 5 years.^19^,^21^ Consequently, MCI can be regarded as a risk state for dementia or a potential precursor to dementia.^15 17^

Livingston *et al* (2024) found evidence that hearing loss is one of the largest potentially modifiable risk factors in mid-life for the development of dementia.^22^ Furthermore, both hearing loss and dementia are age-related conditions that frequently occur together and have several overlapping symptoms.^10 23^ For example, it is common for both individuals living with dementia and individuals living with hearing loss to experience difficulties in following conversations^23^ and increased social withdrawal.^24^ The mechanistic pathways underlying the link between hearing loss and dementia are not yet understood, though several hypotheses have been put forth.^23 25^ For example, the sensory deprivation hypothesis proposes that hearing loss contributes to the development of dementia by causing prolonged auditory deprivation that adversely affects the brain, leading to physical changes in brain structure and function. For instance, auditory deprivation could decrease cortical brain volume and necessitate cortical reorganisation that further restricts the cortical capacity available for cognitive processing. Another example is the information degradation hypothesis, which posits that hearing loss requires increased listening effort and increases demands on cognitive processing to compensate for diminished auditory input and degraded auditory perception. This draws resources away from other higher-level cognitive processing, which could lead to the development of dementia or the earlier presentation of cognitive impairment. The common cause hypothesis proposes that hearing loss and dementia have the same underlying mechanism, such as overall neural degeneration associated with ageing leading to decreased auditory and cognitive ability. Furthermore, the two conditions share several risk factors, particularly vascular risk factors, such as smoking and diabetes.^23 25^

Timely diagnosis of hearing loss in people living with MCI or dementia is important to enable them access support and suitable hearing interventions as early as possible. In the UK, National Institute for Health and Care Excellence (NICE) guidelines state that clinicians should consider referring people with diagnosed MCI or dementia to an audiology service for a hearing assessment every 2 years if they have not previously been diagnosed with hearing loss.^26^ There is evidence that hearing interventions for adults with cognitive impairment are feasible and have the potential to improve dementia-related problem behaviours, communication and social engagement.^27 28^ Further research is needed to determine whether hearing interventions can improve cognition or even help to reduce dementia risk or delay its onset, as studies have produced mixed evidence to date.^29^ In a recent randomised controlled trial, the use of hearing aids decreased cognitive decline over a 3-year period for those at increased risk of developing dementia, though not in those at decreased risk.^30^

Currently, individuals with hearing loss who present to primary care services may have difficulty in obtaining a referral to audiology services. A UK study found that as many as one in five people expressing concerns about hearing loss do not receive a referral for further assessment.^31^ This is potentially due to some healthcare providers having limited knowledge about hearing loss or minimising the impact it may have on the patient.^31 32^ When referral to audiology services for further assessment is achieved, conducting assessments with people living with MCI or dementia may be challenging. Cognitive decline may affect performance on standard audiological tests, including pure-tone audiometry, such as by making it difficult to understand test instructions.^33^ Currently, cognitive testing is not widely integrated into audiological services, although recent qualitative research suggests patients perceive cognitive screening to be acceptable within this setting.^34^ A UK survey reported that, although 90% of audiologists reported a willingness to carry out cognitive testing, fewer than 4% actually do.^35^ Lack of training, time and awareness of cognitive screening tests are barriers to cognitive screening in audiology.^35 36^ Additionally, the provision of hearing aids and other hearing interventions to people living with MCI or dementia may be challenging.^37^ These patients are more likely to experience issues with hearing aid use, such as misplacing them, as well as struggling to autonomously change batteries or to fit and adjust the devices.^38^ Consequently, adaptations to make audiological assessments and interventions more dementia-friendly are recommended.^10 39^

Recommendations have been published for audiologists conducting hearing assessments on patients with diagnosed or suspected MCI/dementia.^33 39^ They include recommendations regarding the physical setting of the consultation (eg, considering whether a home visit is required, ensuring family are present at the appointment), adaptions of standard audiological tests (eg, reducing or increasing test duration), use of alternative audiological tests (eg, cortical evoked potentials) and flexibility in the long-term care of patients with co-occurring dementia and hearing loss (eg, having more frequent follow-up appointments, planning for missed appointments and lost hearing devices). Furthermore, audiologists may adapt the interventions and rehabilitation they provide for people with dementia, including offering hearing aids that are tamper proof, providing written instructions on hearing aid care to be shared with carers and involving professionals from social care, primary care or psychiatry.^10 39^ However, there is currently limited evidence of the widespread application of these recommendations within audiological services.

### Aims and objectives

Though the link between hearing loss and dementia risk is a growing concern, there remains an evident lack of established and widely implemented guidelines in audiology services for assessing and managing patients with suspected or diagnosed comorbid hearing loss and dementia/MCI and for making onward referrals from audiology services to other health services (eg, memory clinics) for these patients.^10^ With an ageing population and the increase in prevalence of these comorbid conditions, there is a clear need to develop an in depth understanding of current practice, including identifying gaps in care that must be addressed to provide beneficial and tailored care to patients. Therefore, this work will comprehensively review the current literature about specialist clinical pathways for patients living with suspected or diagnosed MCI/dementia in audiology services, which will help inform the development best practice guidelines. This review will provide up-to-date information on clinical practice that will benefit patients, carers, clinicians and health services. It will also provide an overview of the current research landscape, identifying gaps in the literature to inform future research hypotheses.

Specifically, the primary aim of this review is to examine whether specialist clinical pathways exist in audiology services for adults living with suspected or diagnosed coexisting hearing loss and MCI/dementia. In this review, specialist clinical pathways refer to all stages and aspects of care given to people living with MCI/dementia and hearing loss in audiology services, including their entry into the service, such as following a referral from a primary care professional, assessment appointments, hearing aid fitting appointments and follow-up and review appointments. Specialist clinical pathways may differ from routine clinical pathways in several ways, such as care being delivered by audiologists with training in dementia, longer appointments for people living with MCI/dementia and collaborations with memory clinics. The objectives of this review are (1) to identify whether specialist clinical pathways for patients with MCI/dementia exist in audiology services; (2) to explore and describe the specific components and features of these pathways, such as specialist training for audiologists, protocols for the onward referral of patients with MCI/dementia from audiology services to other health services (eg, memory clinics) and adapted assessment and management procedures, resources or settings; (3) to understand the similarities and differences between specialist pathways for patients with MCI/dementia and routine pathways for patients without MCI/dementia within audiology services and (4) to examine current practice guidance regarding the provision of audiological care to patients living with MCI/dementia and hearing loss.

## Methods and analysis

This review will be reported in accordance with the Preferred Reporting Items for Systematic Reviews and Meta-Analyses extension for Scoping Reviews (PRISMA-ScR) checklist^40^ and is registered via the Open Science Framework (https://osf.io/7654r).^41^ Any amendments to the protocol will be recorded via the Open Science Framework. The review commenced on 31 January 2024 and is expected to end in May 2025.

### Eligibility criteria

The eligibility criteria are defined according to the domains of the SPIDER search strategy tool, which contains the following domains: Sample, Phenomenon of Interest, Design, Evaluation and Research type. SPIDER is an established alternative to the Population, Intervention, Comparison and Outcomes (PICO) tool and can be applied to qualitative, quantitative and mixed-methods research studies.^42^

### Sample

The sample will be (1) adult patients (aged ≥18, no upper age limit) in audiology services who have suspected or confirmed MCI or dementia, (2) formal carers (eg, care home staff, support workers) and informal carers (eg, relatives, spouses) of patients with suspected or confirmed MCI or dementia who attend audiology services and (3) clinicians who work in audiology services and treat patients with suspected or confirmed MCI or dementia. Both suspected and confirmed MCI or dementia will be included to ensure that the review encompasses as much of the available literature as possible and is inclusive of those without a formal diagnosis of dementia. Research indicates that many people postpone seeking a dementia diagnosis and that there is significant variation across regions and countries in terms of access to an accurate and timely dementia diagnosis.^43^,^45^ The World Alzheimer’s Report (2021) estimated that 75% of all dementia cases go undiagnosed worldwide, which rises to 90% of cases in low-and-middle-income countries.^46^

### Phenomenon of interest

This review will examine current or existing specialist audiology pathways for adults with MCI or dementia. Specifically, this review will examine the existence of these pathways, the components and features of these pathways and their similarities and differences to routine pathways for audiology patients without MCI or dementia. In addition, clinical guidance for the provision of audiological care to patients with MCI or dementia and hearing loss will be examined. Both private and public audiology services will be considered, as well as audiology services embedded in primary care practices, memory clinics and care homes (ie, residential and nursing homes). ENT departments will only be included if specifically discussed within the context of a specialist clinical pathway. All countries will be eligible.

### Design

Primary research studies that produce data relating to the aims and objectives of this scoping review will be considered for inclusion, including studies that collect data via interviews, focus groups, questionnaires and observations. In addition to peer-reviewed articles, the grey literature will also be searched. Specifically, national or international practice guidelines, conference proceedings and conference abstracts will be searched. Professional commentary articles and editorials will be considered for inclusion only if they contain primary data relating to the aims and objectives of this review. The remainder of the grey literature (eg, case reports, case series, magazine articles) will be excluded as this reduces the likelihood of including poor quality studies. Moreover, there is no agreed approach to extracting and synthesising evidence obtained from the grey literature in a transparent way.^47^

### Evaluation

The review will examine the presence of specialist clinical pathways in audiology services for adults with suspected or diagnosed MCI or dementia, their components and features, and how they compare and contrast to routine clinical pathways. This will include examining any guidelines and protocols that are observed, any adjustments made to the assessments and interventions administered to patients, follow-up and referral procedures, outcome measurement, and any specialist dementia training undertaken by clinicians.

### Research type

Published or in-press studies, including qualitative, quantitative and mixed-methods studies, producing data relating to the review aims will be eligible for inclusion. Reviews of other studies (eg, systematic reviews, scoping reviews) will be excluded. Only studies written in English or that have been translated into English will be included because the review team does not have resources available to support translation. The search will be limited to English language publications within the last 15 years, as we are interested in current audiological practices.

### Information sources and search strategy

The following electronic bibliographic databases will be systematically searched by the first author (SMW): CINAHL, EMBASE, MEDLINE, PsycINFO, PubMed, Scopus and Web of Science. Additionally, the OpenGrey archive will be searched for the grey literature. The selection of databases and development of the search strategy were informed by the SPIDER tool and were performed in collaboration with experienced information specialists at the University of Nottingham, in accordance with the PRISMA-ScR checklist.^40^ The proposed search strategy is shown in online supplemental appendix 1.

### Article selection process

All references will be imported into a review management software (eg, Covidence, Rayyan^48 49^), and duplicates will be removed. The first author (SMW) will screen the titles and abstracts of all retrieved articles against the eligibility criteria. A second researcher will independently screen the titles and abstracts of a minimum of 20% of retrieved articles in accordance with AMSTAR (a measurement tool to assess systematic reviews) 2 guidance.^50^ Subsequently, the full text of every potentially relevant article will be obtained. The first author (SMW) will review each full-text article for eligibility. In line with the AMSTAR 2 guidance, a second researcher will independently review at least 20% of the full-text articles. Throughout the review, any disagreements between the first and second researchers will be resolved through discussion. If necessary, a third author will be engaged to make a final decision. In addition, the original authors of the retrieved articles will be approached for additional information and clarification as needed. Reasons for the exclusion of articles will be noted. The search strategy and study selection process will be reported according to the PRISMA-ScR statement, and a PRISMA flow diagram will be presented.

### Data charting and data items

One researcher will extract the data from the included studies. A minimum of 20% of the studies will be randomly selected and checked for consistency by a second researcher. Any discrepancies will be resolved though discussion between the two researchers or, when needed, via consultation with a third researcher and revisiting the original record. Data will be collected using a customised data extraction sheet informed by the SPIDER tool domains: Sample, Phenomena of Interest, Design, Evaluation and Research type^42^ and by the National Institute for Health and Care Excellence (NICE) guidelines for dementia and hearing loss assessment and management.^26 51^ Data extracted will include, but will not be limited to, specific details about the sample characteristics, audiology service characteristics, country, study design and methods and key findings and implications. The data extraction sheet will be continuously modified by the research team throughout data collection in an iterative process, allowing for reflexive analysis of the material.^52 53^ A draft data extraction sheet is available in online supplemental appendix 2.

### Data synthesis

A qualitative synthesis will be performed, which will entail drawing study findings together to enable new explanations and interpretations of the phenomenon of interest to emerge.^54^ The specific methodology employed will be reflexive thematic analysis, conducted in accordance with an established procedure developed by Braun and Clarke.^52 53^ Reflexive thematic analysis is widely used in scoping reviews.^55^,^57^

This approach involves the generation of codes and themes to describe patterns of meaning across the dataset that are salient to the research questions, while also taking into account researcher subjectivity in the knowledge generation process. It comprises six main stages: (1) familiarisation with the dataset; (2) generating initial codes by assigning meaningful labels to extracts of data that represent key concepts or experiences; (3) generating initial themes, which can involve a range of techniques, including creating visual representations (eg, spider diagrams) or organising codes into clusters around a central idea^53^; (4) iteratively reviewing potential themes throughout the data analysis process, including large and small amendments to the themes; (5) defining and naming themes and (6) writing up the findings, including contextualising analyses in relation to existing knowledge and conveying why the data matter and what the findings mean.^58^

QSR International’s NVivo Software (V.14) will support the organisation and analysis of the data. One author will conduct the thematic analysis guided by the six stages above. They will meet regularly with the research team during the data synthesis stage, from the initial familiarisation with the dataset to preparation of the written report. Decisions on codes and themes, as well as the conclusions that can be drawn from these, will be discussed. This process, known as peer debriefing, will help ensure that the analysis is not limited to the perspectives and preconceptions of the first author.

### Quality appraisal

Article quality will be critically appraised using the validated Mixed Methods Appraisal Tool.^59^,^61^ It encompasses five methodological domains: qualitative research, randomised controlled trials, non-randomised studies, quantitative descriptive studies and mixed methods studies. One author will assess the articles selected for data extraction before inclusion in the review. A second author will independently assess 10% of the articles to check for consistency and to resolve any disagreements through discussion. A third author will be consulted if required.

### Patient and public involvement

A panel of patient and public involvement (PPI) representatives, including members of the public with experience of dementia and/or hearing loss and registered audiologists, will contribute to this research. To date, two PPI representatives have confirmed that the topic of this review is important and provided feedback on a summary of this protocol. These representatives are a woman living with hearing loss and a man living with dementia and hearing loss, who are both aged over 60 years. The PPI panel will be asked to assist with data interpretation during this review as well as to contribute to the development of recommendations arising from this review. Once the review is completed, they will be involved in dissemination, especially dissemination targeting healthcare professionals and members of the public.

### Ethics and dissemination

No ethical issues are foreseen in this scoping review. Ethical approval was not required because this is a scoping review and it will entail analysing secondary data that has previously been published. The reporting of this review will be guided by the PRISMA-ScR guidelines. Several dissemination strategies will be employed, including peer-reviewed journal articles, professional magazines for academics and clinicians, national and international academic conferences, social media, and public and patient engagement events and activities.

### Discussion

A protocol is described for a scoping review characterising the literature surrounding specialist clinical pathways in audiology services for adults living with comorbid hearing loss and MCI/dementia. Many people with dementia have hearing loss, which can significantly impact their quality of life and increase carer burden. They may need adaptations and additional support to undergo audiometric testing and to use aural rehabilitation interventions, such as hearing aids.^23^ At present, there is no established and widely implemented guidance for assessing and managing hearing loss within this population. This review will fill an important gap in knowledge by establishing whether specialist clinical pathways exist in audiology services for these patients and characterising their components. The review will also help determine whether there are important gaps in care that must be addressed.

Previous reviews in this area include a systematic review of research studies that evaluated hearing loss interventions in older adults with cognitive impairment.^27^ This contrasts with the present scoping review, which primarily focuses on identifying and describing existing audiology practices for people living with MCI/dementia, not only including intervention practices but also diagnostic practices, specialist training and resources and onward referral procedures. The primary focus on examining existing audiology practices also distinguishes this review from the work of Littlejohn and colleagues, who used literature reviews, surveys, and qualitative and consensus methods to produce interdisciplinary practice recommendations to guide the future development of services and policy for people living with dementia and vision and hearing loss.^33^ Other previous reviews include a narrative review of the literature about dementia and hearing loss, which examined the relationship between the two conditions, the impact of this comorbidity on quality of life and care received, screening and interventions, and opportunities for prevention.^23^ Another narrative review examined alternative hearing care models for older adults who have cognitive impairment, including the integration of hearing healthcare into care already being provided by other geriatric and rehabilitation specialists.^28^ In contrast, the present study will be conducted in accordance with established scoping review guidelines,^40^ including systematically searching the literature, appraising the quality of included studies using an established tool and performing an in-depth qualitative data synthesis. Therefore, the findings of this scoping review will provide novel, in-depth insights on current audiological care for people living with MCI or dementia and hearing loss, which could inform the development and refinement of clinical practice guidelines. This review will also identify gaps in the current literature, which will inform future research studies about this important comorbidity.

## supplementary material

10.1136/bmjopen-2024-087418 online supplemental file 1

10.1136/bmjopen-2024-087418 online supplemental file 2

## Data Availability

Data sharing not applicable as no datasets generated and/or analysed for this study.
