# Supplementary material for: Specialist clinical pathways in audiology services for adults living with coexisting hearing loss and dementia: a scoping review protocol
Source: BMJ Open. 2024 Dec 26;14(12):e087418. doi: 10.1136/bmjopen-2024-087418 (PMC11683898; doi:10.1136/bmjopen-2024-087418)
Supplement: online supplemental file 1 [file bmjopen-14-12-s001.pdf]

Type of review: Scoping

Research question: Specialist clinical pathways in audiology services for adults living with co-existing hearing loss and dementia: A scoping review protocol

Search Strategy

Databases searched:

- CINAHL
- EMBASE
- MEDLINE
- PsycINFO
- PubMed
- Scopus
- Web of Science
- OpenGrey archive

Database		Search strategy
**CINAHL**	#1	exp. dementia/
	#2	exp. hearing loss/
	#3	exp. audiology/
	#4	#1 AND #2 AND #3
	#5	Dementia OR Alzheimer* OR Lewy bod* OR Cognitive decline OR Cognitive impairment OR Cognitive function OR Cognitive disorder OR Memory deficit* OR Memory disorder OR MCI OR Complex patient OR Complex need*
	#6	Hearing loss OR Auditory def* OR Hearing impair* OR Impair* hearing OR Hearing difficult* OR Loss of hearing OR Hearing disorder OR Hearing problem* OR Hearing complaint* OR Hearing dysfunction OR Deaf* OR Hard of hearing OR hearing aid*
	#7	Audiolog* OR Hearing care service OR Hearing health service OR Hearing department OR Hearing clinic OR Hearing service OR Audiologic care OR Aural rehabilitation OR pathway
	#8	#5 AND #6 AND #7
	#9	(#5 OR #1) AND (#6 OR #2) AND (#7 OR #3)
**EMBASE**	#1	exp. dementia/
	#2	exp. hearing loss/
	#3	exp. audiology /
	#4	1 AND 2 AND 3
	#5	Dementia OR Alzheimer* OR Lewy bod* OR Cognitive decline OR Cognitive impairment OR Cognitive function OR Cognitive disorder OR Memory deficit* OR Memory disorder OR MCI OR Complex patient OR Complex need*

	#6	Hearing loss OR Auditory def* OR Hearing impair* OR Impair* hearing OR Hearing difficult* OR Loss of hearing OR Hearing disorder OR Hearing problem* OR Hearing complaint* OR Hearing dysfunction OR Deaf* OR Hard of hearing OR hearing aid*
	#7	Audiolog* OR Hearing care service OR Hearing health service OR Hearing department OR Hearing clinic OR Hearing service OR Audiologic care OR Aural rehabilitation OR pathway
	#8	#5 AND #6 AND #7
	#9	#1 OR #5
	#10	#2 OR #6
	#11	#3 OR #7
	#12	#9 AND #10 AND #11
**MEDLINE and PsychINFO**	#1	exp. dementia/
	#2	exp. hearing loss/
	#3	exp. Audiology/
	#4	#1 AND #2 AND #3
	#5	Dementia OR Alzheimer* OR Lewy bod* OR Cognitive decline OR Cognitive impairment OR Cognitive function OR Cognitive disorder OR Memory deficit* OR Memory disorder OR MCI OR Complex patient OR Complex need*
	#6	Hearing loss OR Auditory def* OR Hearing impair* OR Impair* hearing OR Hearing difficult* OR Loss of hearing OR Hearing disorder OR Hearing problem* OR Hearing complaint* OR Hearing dysfunction OR Deaf* OR Hard of hearing OR hearing aid*
	#7	Audiolog* OR Hearing care service OR Hearing health service OR Hearing department OR Hearing clinic OR Hearing service OR Audiologic care OR Aural rehabilitation OR pathway
	#8	#5 AND #6 AND #7
	#9	#1 OR #5
	#10	#2 OR #6
	#11	#3 OR #7
	#12	#9 AND #10 AND #11
**PubMed**	#1	Dementia [Mesh Major Topic]
	#2	Hearing loss [Mesh Major Topic]
	#3	Audiology [Mesh Major Topic]
	#4	#1 AND #2 AND #3
	#5	Dementia OR Alzheimer* OR Lewy bod* OR Cognitive decline OR Cognitive impairment OR Cognitive function OR Cognitive disorder OR Memory deficit* OR Memory disorder OR MCI OR Complex patient OR Complex need*-title/abstract
	#6	Hearing loss OR Auditory def* OR Hearing impair* OR Impair* hearing OR Hearing difficult* OR Loss of hearing OR Hearing disorder OR Hearing problem* OR Hearing complaint* OR Hearing dysfunction OR Deaf* OR Hard of hearing OR hearing aid* (Title/Abstracts)
	#7	Audiolog* OR Hearing care service OR Hearing health service OR Hearing department OR Hearing clinic OR

		Hearing service OR Audiologic care OR Aural rehabilitation OR pathway (Title/Abstracts)
	#8	#5 AND #6 AND #7
	#9	(#5 OR #1) AND (#6 OR #2) AND (#7 OR #3)
**Scopus**	#1	Dementia (Title/Abstracts/Keywords)
	#2	Hearing loss (Title/Abstracts/Keywords)
	#3	Audiology (Title/Abstracts/Keywords)
	#4	#1 AND #2 AND #3
	#5	Dementia OR Alzheimer* OR Lewy bod* OR Cognitive decline OR Cognitive impairment OR Cognitive function OR Cognitive disorder OR Memory deficit* OR Memory disorder OR MCI OR Complex patient OR Complex need* (Title/Abstracts/Keywords)
	#6	Hearing loss OR Auditory def* OR Hearing impair* OR Impair* hearing OR Hearing difficult* OR Loss of hearing OR Hearing disorder OR Hearing problem* OR Hearing complaint* OR Hearing dysfunction OR Deaf* OR Hard of hearing OR hearing aid* (Title/Abstracts/Keywords)
	#7	Audiolog* OR Hearing care service OR Hearing health service OR Hearing department OR Hearing clinic OR Hearing service OR Audiologic care OR Aural rehabilitation OR pathway (Title/Abstracts/Keywords)
	#8	#5 AND #6 AND #7
	#9	(#5 OR #1) AND (#6 OR #2) AND (#7 OR #3)
**Web of Science**	#1	Dementia (Title/Abstracts/Keywords)
	#2	Hearing loss (Title/Abstracts/Keywords)
	#3	Audiology (Title/Abstracts/Keywords)
	#4	#1 AND #2 AND #3
	#5	Dementia OR Alzheimer* OR Lewy bod* OR Cognitive decline OR Cognitive impairment OR Cognitive function OR Cognitive disorder OR Memory deficit* OR Memory disorder OR MCI OR Complex patient OR Complex need* (Title/Abstracts/Keywords)
	#6	Hearing loss OR Auditory def* OR Hearing impair* OR Impair* hearing OR Hearing difficult* OR Loss of hearing OR Hearing disorder OR Hearing problem* OR Hearing complaint* OR Hearing dysfunction OR Deaf* OR Hard of hearing OR hearing aid* (Title/Abstracts/Keywords)
	#7	Audiolog* OR Hearing care service OR Hearing health service OR Hearing department OR Hearing clinic OR Hearing service OR Audiologic care OR Aural rehabilitation OR pathway (Title/Abstracts/Keywords)
	#8	#5 AND #6 AND #7
	#9	(#5 OR #1) AND (#6 OR #2) AND (#7 OR #3)

**OpenGrey**	#1	Dementia
	#2	Hearing loss
	#3	Audiology
	#4	#1 AND #2 AND #3
	#5	Dementia OR Alzheimer* OR Lewy bod* OR Cognitive decline OR Cognitive impairment OR Cognitive function OR Cognitive disorder OR Memory deficit* OR Memory disorder OR MCI OR Complex patient OR Complex need*
	#6	Hearing loss OR Auditory def* OR Hearing impair* OR Impair* hearing OR Hearing difficult* OR Loss of hearing OR Hearing disorder OR Hearing problem* OR Hearing complaint* OR Hearing dysfunction OR Deaf* OR Hard of hearing OR hearing aid* =
	#7	Audiolog* OR Hearing care service OR Hearing health service OR Hearing department OR Hearing clinic OR Hearing service OR Audiologic care OR Aural rehabilitation OR pathway
	#8	#5 AND #6 AND #7
	#9	(#5 OR #1) AND (#6 OR #2) AND (#7 OR #3)
