# Supplementary material for: Specialist clinical pathways in audiology services for adults living with coexisting hearing loss and dementia: a scoping review protocol
Source: BMJ Open. 2024 Dec 26;14(12):e087418. doi: 10.1136/bmjopen-2024-087418 (PMC11683898; doi:10.1136/bmjopen-2024-087418)
Supplement: online supplemental file 2 [file bmjopen-14-12-s002.pdf]

Supplementary Appendix 2: Draft Data Collection Form

**Review title**

General Information

1. **Date form completed**	
2. **Name/ID of person extracting data**	
3. **Report title**	
4. **Report ID**	
5. **Reference details**	
6. **Publication Type**	
7. **Publication journal**	
8. **Study funding source**	
9. **Possible conflicts of interest**	
10. **Country in which the study was conducted:**	
11. **Notes:**	

Methods

	**Descriptions as stated in the report/paper**
12. **Aim or research question**	
13. **Design**	
14. **Data collection method(s)**	
15. **Data analysis approach**	
16. **Study setting/context**	

Participants

	Description as stated in report/paper
17. **Participant type/category**	
18. **Number of participants**	
19. **Sex**	
20. **Age**	
21. **Other relevant demographics, including whether the participants have MCI, dementia, and/or hearing loss.**	
22. **Notes:**	

Audiology Service Characteristics

	Description as stated in report/paper
23. **Where is the audiology service embedded? (e.g., primary or secondary care practice, memory clinic, care home)**	
24. **Is the audiology practice private or public?**	
25. **Is the audiology service integrated into a care pathway?**	

	**Description as stated in report/paper**
26. **Is the specialist pathway identified as existing or required or both?**	
27. **Who attends the specialist pathway?**	
28. **Describe the key components of specialist pathways.**	
29. **Is there existing or required specialist training for audiologists to deliver the specialist pathway? If so, please describe.**	
30. **Any other key information about the professionals who deliver the specialist pathway?**	
31. **What guidelines and protocols, if any, are in place to deliver the specialist pathway?**	
32. **Are any capacity assessments or cognitive assessments undertaken? If so, please specify**	
33. **Any other adjustments made when treating patients with MCI/dementia and hearing loss**	
34. **What are the outcomes/impact of the specialist pathway for patients, carers, clinicians etc?**	
35. **If reported, what is the standard pathway of the audiology service for patients without dementia, and how does it differ and/or mirror the specialist pathway?**	

	Description as stated in report/paper
36. **Add any relevant figures/diagrams here.**	

Other information

	Description as stated in report/paper
37. **Key conclusions/ recommendations of study authors**	
38. **Strengths of study**	
39. **Weaknesses/Limitations of the study**	
40. **References to other relevant studies**	
41. **If applicable, further study information requested and received from the authors**	
42. **Notes:**	
