# Supplementary material for: Specialist clinical pathways in audiology services for adults living with coexisting hearing loss and dementia: a scoping review protocol
Source: BMJ Open. 2024 Dec 26;14(12):e087418. doi: 10.1136/bmjopen-2024-087418 (PMC11683898; doi:10.1136/bmjopen-2024-087418)
Supplement: Supplementary file 3 [file bmjopen-2024-087418.reviewer_comments.pdf]

PEER REVIEW HISTORY

BMJ Open publishes all reviews undertaken for accepted manuscripts. Reviewers are asked to complete a checklist review form and are provided with free text boxes to elaborate on their assessment. These free text comments are reproduced below.

ARTICLE DETAILS

Title (Provisional)

Specialist clinical pathways in audiology services for adults living with co-existing hearing loss and dementia: A scoping review protocol

Authors

Wenje, Sophie Mai; Calvert, Sian; Henshaw, Helen; Spriggs, Ruth V; Dening, Tom; Hendron, Elizabeth; Heffernan, Eithne

---

VERSION 1 - REVIEW

**Reviewer**	**1**
**Name**	**Cross, Hannah**
**Affiliation**	**The University of Manchester**
**Date**	**30-Apr-2024**
**COI**	**None.**

Thank you for the opportunity to review this manuscript. I really enjoyed reading the protocol which clearly outlines plans for a future scoping review. The protocol is well planned and it is easy to see that a lot of work has gone into designing the review.

The topic of hearing loss in people with dementia, and how best to address this need, is of great importance. The results will aid in developing best practice guidelines for managing hearing needs in people with dementia, for which there are no official guidelines currently. The protocol is of relevance to this journal, it is well written, flows well and I understood the aims and objectives clearly. I have a few minor recommendations to the authors of the paper:

It would be helpful to add 'Mixed Methods Appraisal Tool' following 'Article quality will be appraised using a validated tool.' in the Abstract (page 3).

The introduction is quite long in comparison to the rest of the paper. To avoid the readers starting to skim read the intro, it could be helpful to condense this. For example, I found that the first paragraph on page 6 (lines 1-37) was not of direct relevance to the protocol but is more background information, so could potentially be removed.

Page 10 (line 44): Please provide some justification for including both people with confirmed dementia and suspected dementia. I'm assuming this is to encompass as much available literature as possible, and to include those without a formal diagnosis? Clarification on this would be helpful.

Page 12 (line 52) how many of the titles and abstracts will be screened by the second reviewer? Will this be 20% like the full text stage?

Could a draft Data Extraction table be provided in the appendix? I understand that you will update this as you extract more data, but is there a starting extraction table that could be provided to readers? (Page 13, lines 31-38).

Are you able to provide a short overview of the PPI contributors? A couple of sentences on their demographics e.g. age, gender, stakeholders (person with dementia/ carer/ audiologist etc.), if you already have this group recruited?

The Limitations could be expanded upon. For example, at the moment they read more like facts/ points about the protocol, rather than limitations- Why is the inclusion of grey lit or only English papers limitations? A second sentence on each of the bullet points would help readers, particularly those unfamiliar with scoping reviews.

**Reviewer**	**2**
**Name**	**Powell, Danielle S**
**Affiliation**	**University of Maryland at College Park**
**Date**	**01-May-2024**
**COI**	**None**

The authors present a planned protocol for a scoping review examining specialist clinical pathways in audiology services for those affected by hearing loss and dementia. The intention of the review is to summarize evidence for clinical practice and recommendations. While the topic is timely, the development of the purpose for another review and contribution to the field/science, as well as reviewer concerns for some of the statements made in the introduction to ensure appropriate statements based on science have led the reviewer to recommend major revision. Concerns can be centered on 3 main areas:

- The authors mention the motivation for the proposal and planned analysis is due to limited evidence for guidelines for "established treatment guidelines and referral pathways". It is unclear to the reviewer what is meant by referral pathways when this is used a few times in the manuscript. Please describe via examples or define what they mean by "referral pathways" so that readers had a better understanding of intention.

- Additionally, there are existing publications which review this content, including Littlejohn et al., Mamo et. al. and Hickson et al. among others. It remains unclear how this specific review will uniquely contribute to the literature and clinical practice as written. The authors might consider careful consideration and highlighting of this contribution further

- There are a number of statements in the introduction which, to the reviewer, are over-generalizing or unclear. The authors should consider careful review of the literature and statements made to ensure accurate representation. While some concerns arose throughout the introduction, a few examples include:

1. Statements that it is hard for clinicians to distinguish between hearing loss and dementia (page 4)- while this can occur, it is not common and statements should reflect as such and a reference provided.

2. On page 6, the authors indicate that research shows those with hearing loss perform worse on cognitive tests with verbal instructions. Other methodologically rigorous research by Nichols et al. proved that again, while this can occur, it is not common.

3. the descriptions of MCI and dementia and review of existing mechanistic theories which have been widely published are unclear to the reviewer. The authors might consider rewriting to ensure clarity for the reader.

VERSION 1 - AUTHOR RESPONSE

Reviewer 1 Comments to the Author:

Thank you for the opportunity to review this manuscript. I really enjoyed reading the protocol which clearly outlines plans for a future scoping review. The protocol is well planned and it is easy to see that a lot of work has gone into designing the review.
The topic of hearing loss in people with dementia, and how best to address this need, is of great importance. The results will aid in developing best practice guidelines for managing hearing needs in people with dementia, for which there are no official guidelines currently. The protocol is of relevance to this journal, it is well written, flows well and I understood the aims and objectives clearly.

Thank you for your feedback. We have addressed each of your comments and believe the manuscript has been substantially improved.

I have a few minor recommendations to the authors of the paper:

It would be helpful to add 'Mixed Methods Appraisal Tool' following 'Article quality will be appraised using a validated tool.' in the Abstract (page 3).

We have added this to the abstract (Lines 53-54).

The introduction is quite long in comparison to the rest of the paper. To avoid the readers starting to skim read the intro, it could be helpful to condense this. For example, I found that the first paragraph

on page 6 (lines 1-37) was not of direct relevance to the protocol but is more background information, so could potentially be removed.

We have substantially amended the Introduction section (Lines 73-194). This included condensing several paragraphs by removing background information that is not of direct relevance to the protocol. In particular, we removed information about other healthcare services (e.g., memory clinics) and non-audiological care, as the focus of the manuscript is on audiology services (Lines 122-151).

Page 10 (line 44): Please provide some justification for including both people with confirmed dementia and suspected dementia. I'm assuming this is to encompass as much available literature as possible, and to include those without a formal diagnosis? Clarification on this would be helpful.

We have now added a justification for including people with confirmed and suspected dementia (Lines 214-220). It is important to include people living with suspected dementia to be as comprehensive and inclusive as possible, as there is evidence that many people living with dementia do not receive a timely formal diagnosis.

Page 12 (line 52) how many of the titles and abstracts will be screened by the second reviewer? Will this be 20% like the full text stage?

The second reviewer will screen a minimum of 20% of screened articles (Line 269). This information has now been added to the Methods section.

Could a draft Data Extraction table be provided in the appendix? I understand that you will update this as you extract more data, but is there a starting extraction table that could be provided to readers? (Page 13, lines 31-38).

A draft data extraction form has now been provided in Appendix 2 (Lines 291-292 and Appendix 2).

Are you able to provide a short overview of the PPI contributors? A couple of sentences on their demographics e.g. age, gender, stakeholders (person with dementia/ carer/ audiologist etc.), if you already have this group recruited?

We have not recruited the full PPI panel for the review yet. Demographic information for the two PPI representatives who have contributed to the protocol have now been added (Lines 328-330).

The Limitations could be expanded upon. For example, at the moment they read more like facts/ points about the protocol, rather than limitations- Why is the inclusion of grey lit or only English papers limitations? A second sentence on each of the bullet points would help readers, particularly those unfamiliar with scoping reviews.

We have revised the strengths and limitations section (Lines 59-70) in line with the Editor's comment: "This section should contain up to five short bullet points, no longer than one sentence each, that relate specifically to the methods. The novelty, aims, results or expected impact of the study should not be summarised here." We have expanded on the strengths and limitations within this section in line with your suggestion.

<u>**Reviewer 2 - Comments to the Author:**</u>

The authors present a planned protocol for a scoping review examining specialist clinical pathways in audiology services for those affected by hearing loss and dementia. The intention of the review is to summarize evidence for clinical practice and recommendations. While the topic is timely, the development of the purpose for another review and contribution to the field/science, as well as reviewer concerns for some of the statements made in the introduction to ensure appropriate statements based on science have led the reviewer to recommend major revision.

Thank you for your valuable comments. We have revised the manuscript in accordance with your feedback and believe it has been significantly improved as a result.

Concerns can be centered on 3 main areas:
- The authors mention the motivation for the proposal and planned analysis is due to limited evidence for guidelines for "established treatment guidelines and referral pathways". It is unclear to the reviewer what is meant by referral pathways when this is used a few times in the manuscript. Please describe via examples or define what they mean by "referral pathways" so that readers had a better understanding of intention.

We agree the term "referral pathways" was unclear and have removed it from the manuscript (Lines 162-163, Lines 165-169, and Line 229). We have now clarified that the review focuses on specialist clinical pathways for people living with MCI/dementia and hearing loss within audiology services and provided an explanation of this term (Lines 180-190). We have specified that the referral of patients with MCI/dementia from audiology services to other health services (e.g., memory clinics) is a potential component or feature of the specialist clinical pathways that will be examined in this review (Lines 186-191).

- Additionally, there are existing publications which review this content, including Littlejohn et al., Mamo et. al. and Hickson et al. among others. It remains unclear how this specific review will uniquely contribute to the literature and clinical practice as written. The authors might consider careful consideration and highlighting of this contribution further.

Thank you for this feedback. We have added a section to the manuscript that discusses relevant existing reviews and highlights the unique contribution of the present scoping review (Lines 347-372). In summary, some previous reviews (e.g., Mamo et al. 2017) were narrative reviews or unstructured literature reviews. In contrast, our review will be conducted in accordance with established scoping review guidelines and will entail systematically searching the literature, appraising the quality of included studies using an established tool, and performing an in-depth qualitative data synthesis. Mamo et al. (2018) conducted a systematic review of research studies

that evaluated hearing loss interventions in older adults with cognitive impairment. Our scoping review primarily focuses on identifying and describing existing audiology services for people living with MCI/dementia, including not only interventions but a range of components, such as diagnostic assessments, follow-up care, and specialist training for clinicians. We will establish whether specialist clinical pathways currently exist within audiology services for these patients and provide an in-depth analysis of their components, which will enable us to determine whether there are important gaps in care that must be addressed. This differs from the primary aim of Littlejohn et al. (2022), who used literature reviews, surveys, and qualitative and consensus methods to produce interdisciplinary practice recommendations to guide the future development of services and policy for people living with dementia and vision and hearing loss.

- There are a number of statements in the introduction which, to the reviewer, are over-generalizing or unclear. The authors should consider careful review of the literature and statements made to ensure accurate representation.

We have made substantial amendments to the Introduction (Lines 73-163) based on your comments, as well as the comments from Reviewer 1. We have improved its clarity and ensured accurate representation throughout. We have described the changes made in further details below and highlighted them in the manuscript.

While some concerns arose throughout the introduction, a few examples include:
1. Statements that it is hard for clinicians to distinguish between hearing loss and dementia (page 4)- while this can occur, it is not common and statements should reflect as such and a reference provided.

As part of condensing the Introduction section, these statements have been removed.

2. On page 6, the authors indicate that research shows those with hearing loss perform worse on cognitive tests with verbal instructions. Other methodologically rigorous research by Nichols et al. proved that again, while this can occur, it is not common.

Thank you for this feedback. As part of condensing the Introduction section, this statement has been removed so that we maintain focus on audiology services, rather than other services (e.g., memory clinics, primary care) where cognitive testing typically takes place.

3. the descriptions of MCI and dementia and review of existing mechanistic theories which have been widely published are unclear to the reviewer. The authors might consider rewriting to ensure clarity for the reader.

We have rewritten the descriptions of MCI and dementia (Lines 82-101) to improve their clarity. We have also rewritten the section about potential mechanisms underlying the link between dementia and hearing conditions (Lines 107-121). Reviewer 1 recommended condensing the

Introduction section and minimising content that is not directly relevant to the aims of the review. Therefore, we have provided examples of three hypothesised mechanisms, rather than a comprehensive review of all potential mechanisms.

VERSION 2 - REVIEW

**Reviewer**	**2**
**Name**	**Powell, Danielle S**
**Affiliation**	**University of Maryland at College Park**
**Date**	**20-Nov-2024**
**COI**	

The authors have addressed the reviewers concerns. Thank you for the opportunity to review this manuscript.
